# Malaria Reemergence in Northern Afghanistan

**DOI:** 10.3201/eid1309.061325

**Published:** 2007-09

**Authors:** Michael K. Faulde, Ralf Hoffmann, Khair M. Fazilat, Achim Hoerauf

**Affiliations:** * Central Institute of the Bundeswehr Medical Service, Koblenz, Germany; † Provincial Malaria Unit, Kundoz, Afghanistan; ‡ University Clinic Bonn, Bonn, Germany

**Keywords:** Rice-field malaria, outbreak, Afghanistan, *Plasmodium falciparum*, *Plasmodium vivax*, *Anopheles pulcherrimus*, *Anopheles hyrcanus*, *Anopheles superpictus*, dispatch

## Abstract

Field investigations were conducted in Kundoz Province, an Afghan high-risk area, to determine factors responsible for the rapid reemergence of malaria in that country, where 3 million cases were estimated to have occurred during 2002. Results indicate the presence of nonrice-field–dependent *Plasmodium falciparum* and rice-field–associated *P. vivax* malaria.

In 2002, the total malaria incidence in Afghanistan was estimated to be 3 million cases per year, most of them in Kundoz Province. Field investigations from 2001 through 2005 showed a rapid reemergence of malaria caused by *Plasmodium falciparum* and *P. vivax*, with annual incidence rates from 0.0088 to 4.39 and from 3.58 to 13.37 episodes per 1,000 person-years, respectively. Both diseases peaked during 2002 and then declined independently. Although control campaigns against falciparum malaria, transmitted by the freshwater breeder *Anopheles superpictus*, have been successful, *P. vivax* malaria remains highly endemic and is associated with rice-growing areas, where it is transmitted by the endophilic and exophilic rice-field breeders, *A. pulcherrimus* and *A. hyrcanus*. *P. vivax* polymorph VK 247 prevailed in 90% of infected mosquito pools. Field data showed anthropogenically induced increases in rice-field vivax malaria in northern Afghanistan and the need for further control strategies, including large-scale larval mosquito eradication, in rice-growing areas.

Malaria is endemic to large areas of Afghanistan that are <2,000 meters above sea level, but high-altitude epidemic *P. falciparum* malaria may occur in areas >2,400 meters above sea level (*1*). From the 1950s through 1979, malaria control in Afghanistan was implemented by the government (*2**,**3*). During the 1970s, the number of recorded cases of malaria varied from 40,000 to 80,000 annually (*4*). At that time, vivax malaria chiefly occurred in the irrigated zones of northeastern Afghanistan (*3*). Rice fields were located >5 km away from villages to exceed the flight range of vector-competent, widely DDT-resistant anopheline mosquitoes, and larvivorous *Gambusia affinis* fish were continuously reared and widely introduced (*5**,**6*). After 1980, chronic political instability resulted in the progressive breakdown of malaria control activities (*2*).

Although existing malaria control efforts have focused mainly on the Kabul area, little is known about the situation in the irrigated rice-growing high-risk areas of northeastern Afghanistan (*7*). During 1996–2001, from 202,767 to 395,581 malaria cases were reported annually, sharply increasing in 2002 and 2003 with 590,176 and 591,441 cases confirmed, respectively (*7*), and 3 million cases estimated annually (*8*). Takhar and Kundoz Provinces were most affected (*7*). In late 2003, *P. falciparum* incidence ranged from 0.002% in Wardak to 31% in Takhar Province. The other malaria cases were attributable to *P. vivax* (*7*). Our aim was to analyze the current status, risk factors, and epidemiology of malaria in Kundoz Province, a previously underreported risk area.

## The Study

Newly contracted (excluding all follow-up patients with *P. vivax* relapses) malaria cases were confirmed by light microscopy, using standard Giemsa staining according to the World Health Organization (WHO) national malaria treatment and diagnosis guidelines (*7**–**9*), and were detected passively in febrile patients seeking treatment in the Provincial Malaria Center, Kundoz City, from January 2001 through December 2005. Annual cases of *P. vivax* and *P. falciparum* malaria reported from Kundoz Province from January 2001 through December 2005 are depicted in the Figure. A marked increase in case numbers of both vivax and falciparum malaria occurred from 2001 through 2002, showing an 8.9-fold increase for *P. falciparum.* After 2002, the height of the epidemic, malaria case numbers steadily declined, with *P. vivax* cases falling to 10,946 and *P. falciparum* cases falling to only 27 during 2005. With an estimated population of 3,058,100, the annual incidence rates for *P. falciparum* malaria in Kundoz Province were from 0.0088 (in 2005) to 4.39 (in 2002) per 1,000 person-years; for *P. vivax*, the rates were from 3.57 (in 2005) to 13.37 (in 2002) per 1,000 person-years.

**Figure:**
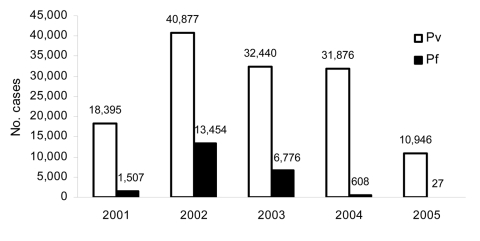
*Plasmodium vivax* (Pv) and *P. falciparum* (Pf) malaria cases reported in Kundoz Province, northern Afghanistan, January 2001–December 2005.

From January 2004 through December 2005, adult anopheline mosquitoes were collected outdoors by using New Standard Miniature Light Traps (No. 1012, John W. Hook Co., Gainesville, FL, USA) without an additional CO_2_ generator and indoors by using an aspirator in the rice-growing areas of Kundoz City, Kanam, Khanabad, Angor Bag, Alchira, Malaghi, and Jan Guzar. Light Traps were set in housing areas within a 5-km radius of rice fields, which are located in or close to towns, villages, and housing areas. Anopheline larval monitoring was carried out using the WHO-recommended Frisbee disk method (*10*) once a month from May through October in rice fields associated with mosquito trapping sites. Results represent mean values obtained after 10 replicates.

Indoor trapping showed the following: of 299 anopheline mosquitoes trapped in 2004, 82.6% were *A. pulcherrimus,* 16.7% were *A. superpictus,* and 0.7% were *A. culicifacies*; of 403 anophelines trapped in 2005, 81.1% were *A. pulcherrimus* and 18.9% were *A. superpictus* (*11*). All specimens were female and blood-fed.

Outdoor entomologic surveys showed the following: of 439 anophelines collected in 2004, 60.1% were *A. pulcherrimus*, 30.0% were *A. hyrcanus*, and 9.9% were *A. superpictus;* of 456 anophelines collected in 2005, 47.4% were *A. hyrcanus*, 42.1% were *A. pulcherrimus*, and 10.5% were *A. superpictus*. Among all mosquitoes trapped, 80.8% were female, and 22.9% of these were blood-fed. The mean trap rate was 4.8 ± 3.9 anophelines per trap night (range 0–17 per trap night).

Anopheline adult outdoor abundance peaked in late August, with the following percentage monthly means: May (1.2%), June (9.5%), July (18.6%), August (35.2%), September (26.8%), and October (8.7%). Anopheline larval monitoring yielded 54.7% *A. hyrcanus* (0–68 larvae per dip; mean 12.3), and 45.3% *A. pulcherrimus* (0–49 larvae per dip; mean 9.8). No *A. superpictus* or *A. culicifacies* larvae could be detected in rice field samples. Anopheline larval abundance peaked in late July and early August with the following monthly means: May (0%), June (17.9%), July (32.3%), August (36.2%), September (12.8%), and October (0.8%).

The *P. falciparum* and *P. vivax* polymorphs VK 210 and VK 247 circumsporozoite protein (CSP) positivity rates in anopheline pools (5 females per species) trapped indoors and outdoors from 2004 through 2005 were detected by using the VecTest Malaria Panel Assay dipstick ELISA (Medical Analysis Systems, Inc., Camarillo, CA, USA) and are listed in the Table. The available data indicate that *A. superpictus* is the principal *P. falciparum* vector. Three *A. pulcherrimus* pools positive for *P. falciparum* CSP indicate that this species may be partly involved in *P. falciparum* malaria transmission. *Plasmodium* CSP positivity values were higher in indoor-trapped *A. superpictus* (2004: χ^2^ = 4.9; df = 1; p = 0.025). Of *P. vivax* CSP-positive pools, 90.6% were VK 247-reactive, and 9.4% were reactive against both VK 247 and VK 210, indicating a similar *P. vivax* genospecies distribution pattern as reported previously from eastern Afghanistan (*12*).

**Table:** Estimated annual malaria incidence rates, Kundoz Province, northern Afghanistan, 2001–2005*

Year	*Plasmodium falciparum* malaria incidence	*P. vivax* malaria incidence	Total malaria incidence
2001	0.49	6.01	6.50
2002	4.39	13.36	17.76
2003	2.21	10.60	12.82
2004	0.19	10.42	10.62
2005	000.88	3.57	3.58

*Cases per 1,000 population.

## Conclusions

Our results show that malaria quickly reemerged in rice-growing Kundoz Province of northeastern Afghanistan. This may be due to various factors: 1) introduction of *P. falciparum* and *P. vivax* malaria by returning refugees (*13*); 2) environmental changes caused by intensified rice growing in close proximity to towns, villages, and housing areas and therefore within flight range of endemic anopheline vectors (*3**,**5*); 3) increased abundance and breeding of the local principal vectors of *P. vivax* malaria stemming from intensified rice growing and irrigation systems that serve as preferred breeding sites for *A. pulcherrimus* and *A. hyrcanus* (*3**,**5*); and 4) absence of widespread biological and chemical vector control measures, including effective larviciding in flooded rice fields (*8*).

Habitat and breeding site preferences of malaria vectors may play a major role in the differing epidemiologies of local *P. falciparum* malaria and rice-field–dependent, exophilic and endophilic *P. vivax* malaria. Possible reasons for the decline in annual malaria cases after 2002, especially in endophilic *P. falciparum* malaria not dependent on rice fields, may include the introduction of insecticide-treated bednets, increased indoor spraying, and improved treatment and health education (*7**,**8*), as well as inhibiting climatic conditions (e.g., the extraordinarily cold 2005 spring/summer season). Current *P. vivax* malaria incidence rates indicate that future control efforts should emphasize large-scale management of potential mosquito breeding sites in rice-growing areas, including biological or chemical larviciding or both. The effectiveness of personal protection from exophilic *P. vivax* malaria vectors such as *A. hyrcanus* may be enhanced by simultaneous use of skin repellents and insecticide-treated clothing (*14**,**15*).
